# New Insights from Nonequilibrium Kinetics Studies on Highly Polar *S*-Methoxy-PC Infiltrated into Pores

**DOI:** 10.1021/acs.jpclett.2c02672

**Published:** 2022-11-03

**Authors:** Magdalena Tarnacka, Ewa Kamińska, Marian Paluch, Kamil Kamiński

**Affiliations:** † Institute of Physics, University of Silesia in Katowice, 75 Pulku Piechoty 1, 41-500Chorzow, Poland; ‡ Department of Pharmacognosy and Phytochemistry, Faculty of Pharmaceutical Sciences in Sosnowiec, Medical University of Silesia in Katowice, Jagiellońska 4, 41-200Sosnowiec, Poland

## Abstract

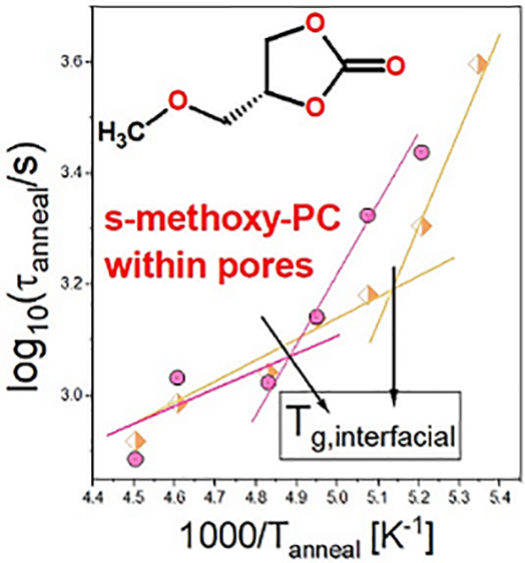

Herein, the annealing of highly polar (*S*)-(−)-4-methoxymethyl-1,3-dioxolan-2-one (*S*-methoxy-PC) within alumina and silica porous membranes characterized by different pore diameters was studied by means of dielectric spectroscopy. We found a significant slowing down of the structural dynamics of confined *S*-methoxy-PC with annealing time below and, surprisingly, also above the glass transition temperatures of the interfacial layer, *T*_g,interfacial_. Furthermore, unexpectedly, a change in the slope of temperature dependencies of the characteristic time scale of this process *τ*_anneal_, at *T*_g,interfacial_ for all confined samples, was reported. By modeling *τ*_anneal_(*T*), we noted that the observed enormous variation of *τ*_anneal_ results from a decrease of the pore radius due to the vitrification of the interfacial molecules. This indicates that the enhanced dynamics of confined materials upon cooling is mainly controlled by the interfacial molecules.

Soft materials constrained at the nanoscale have gained increasing attention due to their unique and confinement-dependent properties. Importantly, although correlations between the kind of spatial restriction, finite size effects, surface interactions, and final features of nanomaterials have been intensively studied for a few decades, there are still many unsolved issues that have yet to be addressed. Especially crucial and puzzling seems to be the change in the diffusion/structural and segmental dynamics observed for different glass formers, infiltrated into mesoporous membranes.^1−6^ In those cases, the structural/segmental relaxation times, *τ*_α_, of confined materials deviate from the bulk behavior at a specific temperature, higher than the glass transition temperature, *T*_g_, of the macroscale sample. Recently, a few possible explanations for this finding have been proposed.

At first, such behavior was often discussed in terms of the changes in the temperature evolution of dynamical heterogeneities within the glass formers constrained at the nanoscale. Briefly, the pronounced slowing down of supercooled liquid dynamics is considered to be a result of an increasing length scale of cooperatively rearranging regions (ξ).^7^ Thus, it was assumed that, in the case of materials infiltrated into mesopores, ξ cannot increase due to restriction posed by the pore size, leading to the deviation of *τ*_α_. Taking into account the size of ξ, which is roughly estimated as ξ ∼2 nm, the above-mentioned rationalization should be valid only for the templates having pore diameters, *d*, similar to ξ.^1,2,8−12^ However, the deviation of *τ*_α_(*T*) dependencies from the bulk behavior near *T*_g_ was also frequently reported for glass formers infiltrated within mesoporous membranes of significantly higher pore sizes, *d* > 18 nm,^3,6,13^ questioning the correlation between dynamical heterogeneities and the enhanced dynamics of confined liquids. The other approach links the change in mobility of the nanospatially restricted systems to the occurrence of the spinodal temperature.^14,15^ Nevertheless, this effect seems to be only limited to polymeric materials characterized by high molecular weights, as for medium- and low-molecular-weight glass formers infiltrated within anodic aluminum oxide (AAO) templates, the differences in the molecular dynamics between bulk and confined materials were reported independently from the applied thermal protocol.^16^

Lastly, the deviation of confined *τ*_α_(*T*) dependencies has been discussed in terms of the surface effects. Interestingly, the combination of dielectric and calorimetric data revealed that the temperature at which the change in *τ*_α_ occurs agrees with the calorimetric glass transition temperature assigned to the vitrification of the interfacial molecules (adsorbed on the pore walls, *T*_g,interfacial_).^3^ According to this hypothesi,s below *T*_g,interfacial_, immobilized adsorbed molecules generate pseudoisochoric conditions, where free volume barely varies, resulting in a change in the temperature dependence of structural relaxation times that mimics *τ*_α_(*T*) evolution at constant volume.^17,18^ Finally, one should also mention recent studies that clearly showed that roughness-induced variation in the mobility of adsorbed molecules strongly affects the overall dynamics of polymer films,^19^ which agrees with the results of molecular dynamics simulation studies.^20−22^ Nevertheless, surprisingly, the chemical modification of the inner pore walls (leading to the change in the strength of interfacial interactions) does not influence *T*_g,interfacial_.^23^ This data increases doubt about the vitrification of the interfacial layer being responsible for a change in the molecular dynamics of confined materials.

To gain deeper insight into this problem, we carried out a series of annealing experiments in the close vicinity of *T*_g,interfacial_ on (*S*)-(−)-4-methoxymethyl-1,3-dioxolan-2-one (*S*-methoxy-PC, see the chemical structure in the inset in Figure 1a),^24,25^ incorporated into both AAO and silica porous templates characterized by different surface energy, wettability, and pore diameters, *d*, using broadband dielectric spectroscopy (details are shown in the Supporting Information). It should be highlighted that, up to now, the vast majority of such annealing experiments were performed only in a limited range of temperatures (*T* < *T*_g,interfacial_), as above *T*_g,interfacial_, the effects of isothermal time-dependent measurements were hardly detectable. It is also worth mentioning that *S*-methoxy-PC (static permittivity, ε_s_ ∼180)^24,25^ was explicitly chosen due to strong dipole–dipole interactions and interesting effects (i.e., two-step aging) accompanying equilibration of this compound below *T*_g_.^26−28^

**Figure 1:**
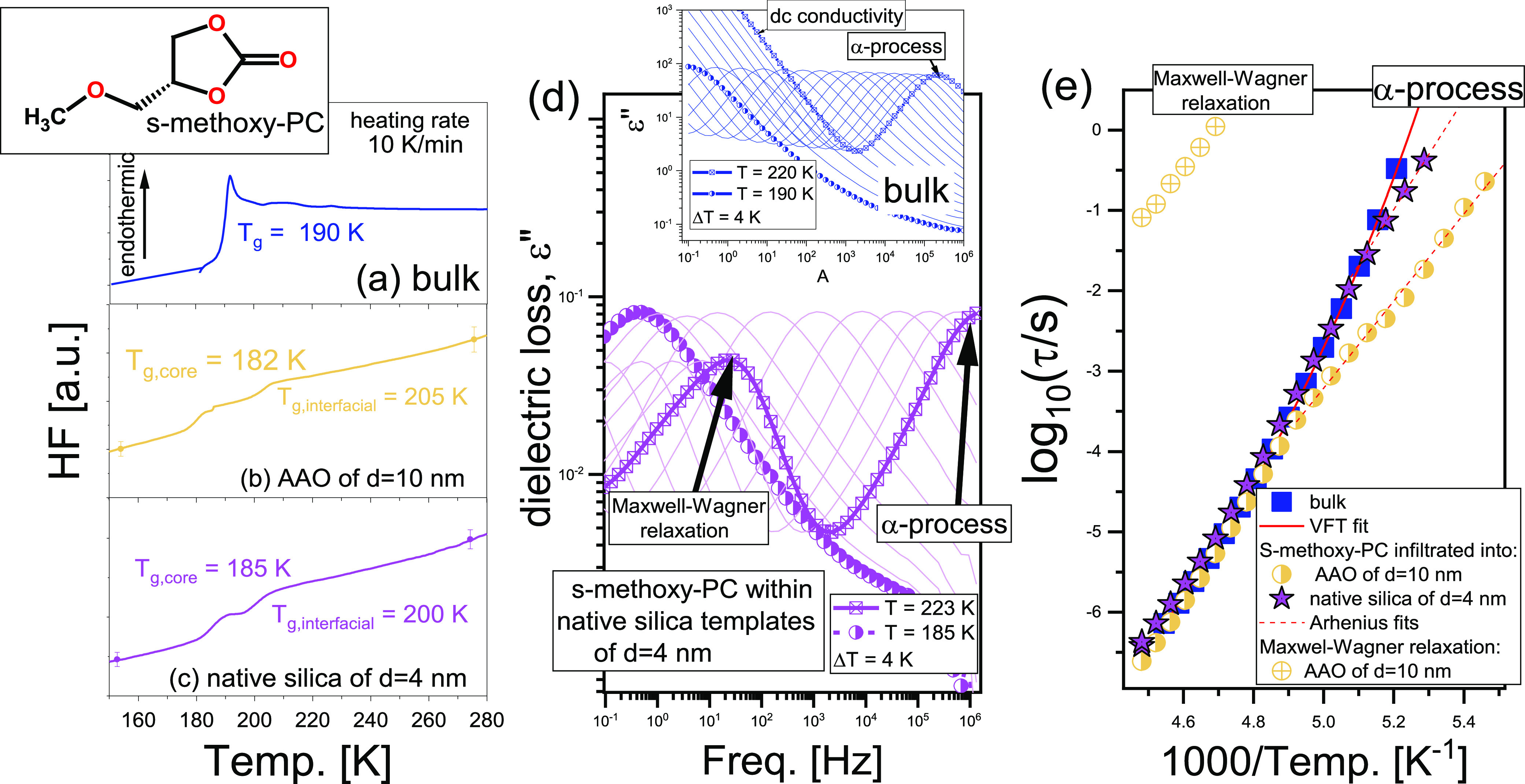
DSC curves of bulk *S*-methoxy-PC (a) and the infiltrated samples (b, c). The inset in panel a shows the chemical structure of the investigated substance. (d) Representative loss spectra of *S*-methoxy-PC within native silica templates of *d* = 4 nm measured above *T*_g,core_. The inset in panel d illustrates the loss spectra of the bulk sample. (e) *τ*_α_(*T*) determined for bulk and infiltrated *S*-methoxy-PC. Red lines are the best fits to VFT (solid) and Arrhenius (dashed) equations (details shown in the Supporting Information).

Thermograms obtained for bulk *S*-methoxy-PC and the sample incorporated within various mesoporous membranes are shown in Figure 1a–c. As illustrated, both spatially restricted materials exhibit two glass transitions, located above and below the single one observed for the macroscale system. According to the literature,^30,31^ this phenomenon occurs as a result of internal heterogeneity of the confined liquids where, due to additional interactions with the template, one can distinguish various molecules labeled as “core” (in the center of nanochannels) and interfacial (adsorbed/interacting with the pore walls).^32^ Interestingly, we do not observe significant differences in *T*_g_’s between confined samples irrespectively of either pore radius or wettability (quantified by the contact angle, θ, Table 1). Note that all calorimetric glass transition temperatures, *T*_g_s, were estimated as the midpoints of the step in specific heat measured on heating runs. In fact, previous studies have revealed that there is also no difference in both *T*_g_s and the length scale of the interfacial layer^33^ (ζ ∼ 0.5 nm) for *S*-methoxy-PC incorporated into native and silanized silica templates having comparable *d* and much different contact angles.^29^ This indicates that the behavior of the highly polar liquid infiltrated into porous membranes is not governed by wettability but rather by other factors, probably the density packing and the lower extent of guest–host interactions. Importantly, similar effects have been reported for poly(methylphenylsiloxane) (PMPS) within AAO templates.^23^

**Table 1:** Values of θ and *γ*_SL_ at *T* = 298 K Determined for Investigated Systems^a^

surface	contact angle, θ [deg]	interfacial energy, *γ*_SL_ [mN/m]
alumina	29.2	21.4
native silica	40.0	34.6

a Data was taken from ref (29).

Furthermore, complementary dielectric measurements performed on the bulk sample showed the presence of the two relaxation processes: (i) the dc conductivity and (ii) the α-relaxation (related to the glass transition phenomenon) at low and high frequencies, respectively (inset in Figure 1d). In the case of infiltrated materials, apart from the charge transport and the structural mode, also the Maxwell–Wagner (MW) polarization at the middle-frequency range can be seen (Figure 1d).^34^ The analysis of the dielectric loss spectra revealed that all spatially restricted samples exhibit a pronounced deviation from the structural dynamics of the bulk material (see Figure 1e). Note that the dielectric spectra were analyzed by the Havriliak–Negami (HN) function^35^ (details are shown in the Supporting Information). Interestingly, *τ*_α_(*T*) obtained for confined samples starts to deviate from the bulk behavior at a temperature in close proximity (within a few K) to *T*_g,interfacial_ determined from calorimetric investigations. Intriguingly, the most prominent departure of the structural dynamics and *T*_g_ from the macroscale *S*-methoxy-PC was noted for the sample infiltrated within AAO templates of *d* = 10 nm. One can recall that, generally, *T*_g,interfacial_ increases with a reduction of the pore size.^3,6^ However, unexpectedly, herein, this effect does not correlate with the pore diameter, θ, or the interfacial energy, *γ*_SL_, questioning in some sense the hypothesis about vitrification of the interfacial layer being responsible for the deviation in structural dynamics of the confined *S*-methoxy-PC from the bulk behavior.^29^

Having this puzzling finding in mind, afterward, we carried out a series of annealing experiments in the close vicinity of *T*_g,interfacial_ to gain additional information about the variation of structural dynamics from the bulklike behavior. As reported in the majority of cases, the prolonged annealing of glass formers at nanoscale confinement^36−38^ results in continuous changes, reflected in retardation of structural/segmental dynamics due to a variation in the density packing at the interface that is transferred to the “core” molecules (of more bulklike dynamics).^39−41^ Interestingly, for some materials, it was even possible to recover the bulklike characteristics with annealing.^9,42,43^ This time-induced variation of the molecular dynamics was assigned to the adsorption–desorption process (leading to the reduction of free volume at the interface)^36,44^ or, alternatively (additionally), to a “mass exchange” between the interfacial fraction and those molecules located at some distance from the surface.^1,9,45,46^ Note that the rearrangement of confinement-induced molecular conformations^47^ might also contribute to the mentioned phenomenon. Recently, it was postulated that, in the case of liquids infiltrated into mesopores, this process is strictly driven by the vitrified interfacial layer, whereas the equilibration time scale correlates with the viscous flow under confinement^43^ and also depends on the pores’ morphology (including their finite size and functionality).^48^ Herein, one can mention the studies on thin polymer films, which also indicated that the rate of the equilibration process of macromolecules under confinement is governed by the dynamics of polymer chains, which are irreversibly adsorbed at the interface.^49^ Importantly, aside from the density fluctuations, Song et al.^50^ also showed that the slow mode (SAP) appearing in the loss spectra collected for the polymeric thin films may drive glasses toward equilibrium. The authors postulated that the dynamical parameter of SAP can be used to predict time scales of equilibration processes taking place both in bulk and under nanoconfinement.^50^

In the performed series of annealing experiments, the quenched samples were reheated to the various annealing temperature, *T*_anneal_, and measured as a function of time. Analogical experiments were carried out for the bulk *S*-methoxy-PC to exclude any quenching effects on data obtained for the confined materials (Figure S1 in the Supporting Information). It should be mentioned that we carried out these studies in a wide temperature range (*T*_anneal_ = 187–212 K), both below and above *T*_g,interfacial_. Interestingly, as shown in Figure 2a, the conducted annealing experiments are accompanied by a shift in the dielectric α-loss peak of *S*-methoxy-PC incorporated into native silica membranes characterized by *d* = 4 nm. Remarkably, a variation in the position of the α-loss peak can be easily observed even at *T*_anneal_ = 207 K (>*T*_g,interfacial_). A comparable shift of the α-mode upon the annealing was also seen for *S*-methoxy-PC infiltrated into AAO of *d* = 10 nm (data not shown). It should be noted that the change in the position of the structural relaxation process with time is not as significant as in the case of both PPG and triphenyl phosphite (TPP) incorporated into AAO templates of various pore sizes (*d* = 18–150 nm), where a variation in the peak position was larger than a decade.^13,16^

**Figure 2:**
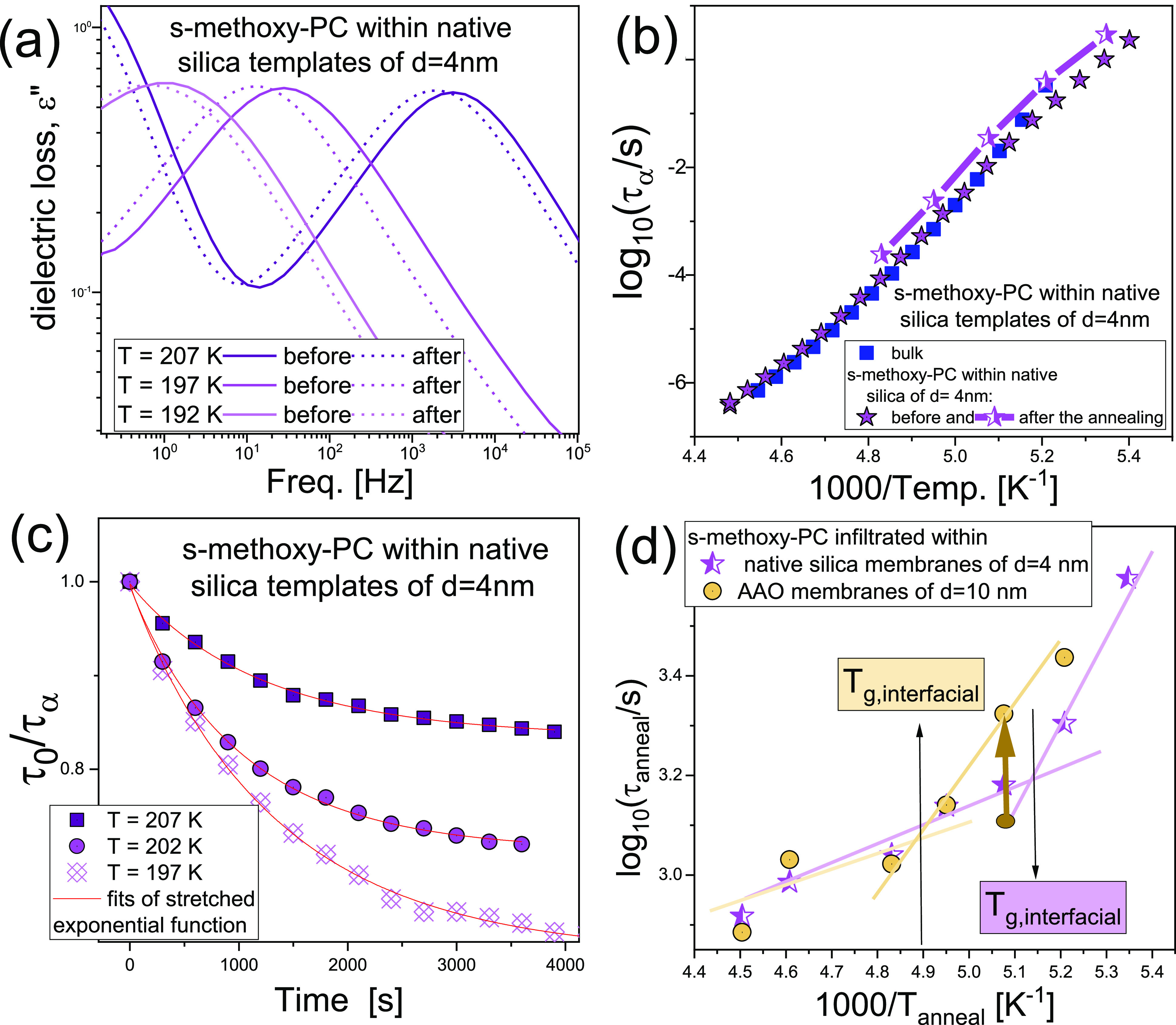
(a) Dielectric loss spectra of *S*-methoxy-PC incorporated into native silica templates of *d* = 4 nm measured before and after the annealing at various *T*_anneal_. (b) *τ*_α_(*T*) of bulk *S*-methoxy-PC and the sample infiltrated into native silica templates of *d* = 4 nm before and after the annealing. (c) Time dependence of τ_0_/*τ*_α_ for *S*-methoxy-PC within native silica templates of *d* = 4 nm. (d) *τ*_anneal_(*T*) determined for the structural process from eq 1.

Immediately after the finished annealing experiments performed at *T*_anneal_ = 207 K, the sample within native silica templates of *d* = 4 nm was quenched (down to 163 K) and remeasured on heating. Figure 2b shows temperature dependencies of *τ*_α_ of bulk *S*-methoxy-PC and the sample infiltrated into native silica membranes of *d* = 4 nm measured both before and after the conducted time-dependent experiments. As illustrated, *τ*_α_(*T*) obtained for the annealed sample significantly differs from those determined before the time-dependent measurements were carried out. Surprisingly, α-relaxation times of confined *S*-methoxy-PC are even slower than those obtained for the bulk substance in the examined temperature range. Consequently, due to the annealing process, we received a nanomaterial of significantly different dynamics, when compared to both bulk and freshly prepared confined samples. It should be emphasized that, to the best of our knowledge, this is the first report demonstrating such a pattern of behavior for liquid incorporated into porous materials. One can recall that, up to now, the recovery of bulk dynamics (including *T*_g_) due to prolonged annealing time was also reported for, i.e., bilayer polystyrene thin films,^51^ bisphenol A diglycidyl ether,^42^ and PMPS^43^ infiltrated in AAO templates. It was assumed that the recovery of bulklike behavior in the case of porous nanomaterials might result from an increase in the density packing of molecules loosely attached to the surface upon annealing.

To explore in detail the equilibration kinetics, the determined *τ*_α_ values changing upon the annealing were rescaled to the initial value, τ_0_, and analyzed with the following equation to determine the equilibration constant of the annealing process, *τ*_anneal_:^52,53^
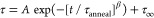
1where *A*, β, and τ_∞_ are constants. Note that, to obtain *τ*_α_ from the isothermal time-dependent dielectric spectra, they were again fitted to the HN function. Representative time dependencies of rescaled α-relaxation times for *S*-methoxy-PC infiltrated into native silica membranes of *d* = 4 nm are shown in Figure 2c. At this point, it should be highlighted that the values of the β parameter from the global fitting reach β = 0.84 ± 0.02 and β = 0.96 ± 0.02 for *S*-methoxy-PC incorporated into AAO of *d* = 10 nm and native silica templates of *d* = 4 nm, respectively. Remarkably, those values are comparable to the *β*_KWW_ parameter, which characterizes the shape of the *bulk* structural relaxation (*β*_KWW_ = 0.84).^24^ Note that, according to the Kohlrausch–Williams–Watts (KWW) function,^54^ the stretched exponent, *β*_KWW_, describes the α-relaxation time distribution’s nonexponentiality. Thus, taking into account the analogy to the aging dynamics,^52^ one can assume that *the structural dynamics governs* the annealing of *S*-methoxy-PC under confinement.

Figure 2d shows the temperature dependencies of determined *τ*_anneal_ for both confined systems. As illustrated, the obtained *τ*_anneal_ increases with a decreasing temperature of annealing. However, there is a clear change in the slope of *τ*_anneal_(*T*) for the samples infiltrated into AAO and native silica membranes. Surprisingly, temperatures, at which this “kink” occurs, agree well with the determined *T*_g,interfacial_ (black arrows in Figure 2d). It should be stressed that the same scenario can be seen in the case of *S*-methoxy-PC incorporated into both AAO templates of *d* = 10 nm and native silica membranes of *d* = 4 nm. This might imply that the vitrification of the molecules interacting with the pore walls strongly affects the processes occurring at the interface, resulting in significantly different slopes of *τ*_anneal_(*T*), below and above *T*_g,interfacial_.

Lastly, one can recall that recent annealing experiments on PMPS infiltrated in AAO templates^43^ have revealed that there is a coupling between the annealing time constant, *τ*_anneal_, and the characteristic viscous flow rate, *τ*_flow_. It was discussed that the faster dynamics of infiltrated polymers is “released through the extremely slow flow, which eventually helps to eliminate surplus volume gained below *T*_g,interfacial_.”^43^ In this context, *τ*_flow_ along nanochannels can be calculated by applying Poiseuille’s law:^55^
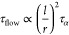
2where *r* is the pore radius, and *l* is the pore length, whereas the viscosity in the original equation is approximated by the structural relaxation times, *τ*_α_, measured by dielectric spectroscopy. Having this in mind, we decided to use eq 2 in order to estimate how much the pore radius must change, Δ*r*, to see a strong deviation in *τ*_anneal_(*T*) (see the dark yellow arrow in Figure 2d). Note that it was assumed that *τ*_anneal_ ∼ *τ*_flow_. Importantly, we found that the observed enormous increase of *τ*_anneal_ from the linear temperature dependence is due to the change of pore radius Δ*r* ∼30%. Interestingly, obtained Δ*r* are in perfect agreement with the values of the length scale of the interfacial layer, ζ, determined from calorimetric measurements.^29,33^ The ζ parameter reaches ζ ∼0.5 nm and ζ ∼1.4 nm for *S*-methoxy-PC infiltrated into silica and AAO templates, respectively (details are shown in the Supporting Information). Thus, one can claim that the significant change in the slope of *τ*_anneal_(*T*) at *T*_g,interfacial_ can be solely explained by the reduction of *r* due to the vitrification of the interfacial layer. Consequently, this also indicates that the faster dynamics of porous materials near *T*_g_ is related to the behavior of the interfacial molecules.

In this paper, we observed that (i) the annealing of incorporated *S*-methoxy-PC is governed by the structural dynamics, and (ii) the determined time scale of the annealing process, *τ*_anneal_, is clearly temperature-dependent. Surprisingly, *τ*_anneal_(*T*) revealed a pronounced change in the slope occurring at the glass transition temperatures of the interfacial layer, *T*_g,interfacial_. This feature was detected for both *S*-methoxy-PC, i.e., infiltrated into AAO of *d* = 10 nm, and native silica membranes of *d* = 4 nm, irrespective of their different pore diameter and wettability. This might imply that the vitrification of the interfacial subset affects (limits) processes occurring at the interface and suppressed mass exchange between interfacial and core molecules. The presented data emphasizes the key role of the interfacial molecules on the deviation of the structural/segmental relaxation times of confined materials from the bulk dynamics.

## Supporting Information Available

Additional experimental details, materials, methods (including sample preparation), as well as time evolution of the loss spectra measured for bulk *S*-methoxy-PC at various *T*_anneal_ (PDF)

## References

[ref1] Arndt M.; Stannarius R.; Groothues H.; Hempel E.; Kremer F. Length Scale of Cooperativity in the Dynamic Glass Transition. Phys. Rev. Lett. 1997, 79 (11), 2077–2080. 10.1103/PhysRevLett.79.2077.

[ref2] Schönhals A.; Stauga R. Dielectric Normal Mode Relaxation of Poly(Propylene Glycol) Melts in Confining Geometries. J. Non. Cryst. Solids 1998, 235–237, 450–456. 10.1016/S0022-3093(98)00657-7.

[ref3] Adrjanowicz K.; Kolodziejczyk K.; Kipnusu W. K.; Tarnacka M.; Mapesa E. U.; Kaminska E.; Pawlus S.; Kaminski K.; Paluch M. Decoupling between the Interfacial and Core Molecular Dynamics of Salol in 2D Confinement. J. Phys. Chem. C 2015, 119 (25), 14366–14374. 10.1021/acs.jpcc.5b01391.

[ref4] Kipnusu W. K.; Elsayed M.; Krause-Rehberg R.; Kremer F. Glassy Dynamics of Polymethylphenylsiloxane in One- and Two-Dimensional Nanometric Confinement - A Comparison. J. Chem. Phys. 2017, 146 (20), 203302 10.1063/1.4974767. 28571386

[ref5] Kipnusu W. K.; Elmahdy M. M.; Elsayed M.; Krause-Rehberg R.; Kremer F. Counterbalance between Surface and Confinement Effects As Studied for Amino-Terminated Poly(Propylene Glycol) Constraint in Silica Nanopores. Macromolecules 2019, 52 (4), 1864–1873. 10.1021/acs.macromol.8b02687.

[ref6] Ananiadou A.; Papamokos G.; Steinhart M.; Floudas G. Effect of Confinement on the Dynamics of 1-Propanol and Other Monohydroxy Alcohols. J. Chem. Phys. 2021, 155 (18), 184504 10.1063/5.0063967. 34773957

[ref7] Gibbs J. H.; Adam G. On the Temperature Dependence of Cooperative Relaxation Properties in Glass-Forming Liquids. J. Chem. Phys. 1965, 43 (2), 139–146. 10.1063/1.1696442.

[ref8] Uhl M.; Fischer J. K. H.; Sippel P.; Bunzen H.; Lunkenheimer P.; Volkmer D.; Loidl A. Glycerol Confined in Zeolitic Imidazolate Frameworks: The Temperature-Dependent Cooperativity Length Scale of Glassy Freezing. J. Chem. Phys. 2019, 150 (2), 024504 10.1063/1.5080334. 30646699

[ref9] Arndt M.; Stannarius R.; Gorbatschow W.; Kremer F. Dielectric Investigations of the Dynamic Glass Transition in Nanopores. Phys. Rev. E - Stat. Physics, Plasmas, Fluids, Relat. Interdiscip. Top. 1996, 54 (5), 5377–5390. 10.1103/PhysRevE.54.5377. 9965723

[ref10] Schönhals A.; Stauga R. Broadband Dielectric Study of Anomalous Diffusion in a Poly(Propylene Glycol) Melt Confined to Nanopores. J. Chem. Phys. 1998, 108 (12), 5130–5136. 10.1063/1.475918.

[ref11] Schönhals A.; Goering H.; Schick C.; Frick B.; Zorn R. Glassy Dynamics of Polymers Confined to Nanoporous Glasses Revealed by Relaxational and Scattering Experiments. Eur. Phys. J. E 2003, 12 (1), 173–178. 10.1140/epje/i2003-10036-4. 15007697

[ref12] Brás A. R.; Merino E. G.; Neves P. D.; Fonseca I. M.; Dionísio M.; Schönhals A.; Correia N. T. Amorphous Ibuprofen Confined in Nanostructured Silica Materials: A Dynamical Approach. J. Phys. Chem. C 2011, 115 (11), 4616–4623. 10.1021/jp107631m.

[ref13] Tarnacka M.; Kaminski K.; Mapesa E. U.; Kaminska E.; Paluch M. Studies on the Temperature and Time Induced Variation in the Segmental and Chain Dynamics in Poly(Propylene Glycol) Confined at the Nanoscale. Macromolecules 2016, 49 (17), 6678–6686. 10.1021/acs.macromol.6b01237.

[ref14] Politidis C.; Alexandris S.; Sakellariou G.; Steinhart M.; Floudas G. Dynamics of Entangled Cis −1,4-Polyisoprene Confined to Nanoporous Alumina. Macromolecules 2019, 52 (11), 4185–4195. 10.1021/acs.macromol.9b00523.

[ref15] Glor E. C.; Angrand G. V.; Fakhraai Z. Exploring the Broadening and the Existence of Two Glass Transitions Due to Competing Interfacial Effects in Thin, Supported Polymer Films. J. Chem. Phys. 2017, 146 (20), 203330 10.1063/1.4979944. 28571332

[ref16] Tarnacka M.; Kaminska E.; Kaminski K.; Roland C. M.; Paluch M. Interplay between Core and Interfacial Mobility and Its Impact on the Measured Glass Transition: Dielectric and Calorimetric Studies. J. Phys. Chem. C 2016, 120 (13), 7373–7380. 10.1021/acs.jpcc.5b12745.

[ref17] Adrjanowicz K.; Kaminski K.; Koperwas K.; Paluch M. Negative Pressure Vitrification of the Isochorically Confined Liquid in Nanopores. Phys. Rev. Lett. 2015, 115 (26), 1–5. 10.1103/PhysRevLett.115.265702. 26765007

[ref18] Tarnacka M.; Kipnusu W. K.; Kaminska E.; Pawlus S.; Kaminski K.; Paluch M. The Peculiar Behavior of the Molecular Dynamics of a Glass-Forming Liquid Confined in Native Porous Materials-the Role of Negative Pressure. Phys. Chem. Chem. Phys. 2016, 18 (34), 23709–23714. 10.1039/C6CP03923E. 27510859

[ref19] Panagopoulou A.; Rodríguez-Tinoco C.; White R. P.; Lipson J. E. G.; Napolitano S. Substrate Roughness Speeds Up Segmental Dynamics of Thin Polymer Films. Phys. Rev. Lett. 2020, 124 (2), 027802 10.1103/PhysRevLett.124.027802. 32004047

[ref20] Mukherji D.; Müser M. H. Glassy Dynamics, Aging in Mobility, and Structural Relaxation of Strongly Adsorbed Polymer Films: Corrugation or Confinement?. Macromolecules 2007, 40 (5), 1754–1762. 10.1021/ma0627370.

[ref21] Hanakata P. Z.; Pazmiño Betancourt B. A.; Douglas J. F.; Starr F. W. A Unifying Framework to Quantify the Effects of Substrate Interactions, Stiffness, and Roughness on the Dynamics of Thin Supported Polymer Films. J. Chem. Phys. 2015, 142 (23), 234907 10.1063/1.4922481. 26093579

[ref22] Hanakata P. Z.; Douglas J. F.; Starr F. W. Interfacial Mobility Scale Determines the Scale of Collective Motion and Relaxation Rate in Polymer Films. Nat. Commun. 2014, 5 (1), 4163 10.1038/ncomms5163. 24932594

[ref23] Winkler R.; Chat K.; Unni A. B.; Dulski M.; Laskowska M.; Laskowski L.; Adrjanowicz K. Glass Transition Dynamics of Poly(Phenylmethylsiloxane) Confined within Alumina Nanopores with Different Atomic Layer Deposition (ALD) Coatings. Macromolecules 2022, 55 (8), 3208–3220. 10.1021/acs.macromol.2c00311.

[ref24] Jedrzejowska A.; Ngai K. L.; Paluch M. Modifications of Structure and Intermolecular Potential of a Canonical Glassformer: Dynamics Changing with Dipole–Dipole Interaction. J. Phys. Chem. A 2016, 120 (44), 8781–8785. 10.1021/acs.jpca.6b08128. 27759381

[ref25] Jedrzejowska A.; Wojnarowska Z.; Adrjanowicz K.; Ngai K. L.; Paluch M. Toward a Better Understanding of Dielectric Responses of van Der Waals Liquids: The Role of Chemical Structures. J. Chem. Phys. 2017, 146 (9), 094512 10.1063/1.4977736.

[ref26] Wojnarowska Z.; Paluch M. Two-Step Aging of Highly Polar Glass. J. Phys. Chem. Lett. 2021, 12 (49), 11779–11783. 10.1021/acs.jpclett.1c03572. 34855403 PMC8686112

[ref27] Körber T.; Stäglich R.; Gainaru C.; Böhmer R.; Rössler E. A. Systematic Differences in the Relaxation Stretching of Polar Molecular Liquids Probed by Dielectric vs Magnetic Resonance and Photon Correlation Spectroscopy. J. Chem. Phys. 2020, 153 (12), 124510 10.1063/5.0022155. 33003722

[ref28] Moch K.; Münzner P.; Böhmer R.; Gainaru C. Molecular Cross-Correlations Govern Structural Rearrangements in a Nonassociating Polar Glass Former. Phys. Rev. Lett. 2022, 128 (22), 228001 10.1103/PhysRevLett.128.228001. 35714246

[ref29] Tarnacka M.; Geppert-Rybczyńska M.; Dulski M.; Grelska J.; Jurkiewicz K.; Grzybowska K.; Kamiński K.; Paluch M. Local Structure and Molecular Dynamics of Highly Polar Propylene Carbonate Derivative Infiltrated within Alumina and Silica Porous Templates. J. Chem. Phys. 2021, 154 (6), 064701 10.1063/5.0040150. 33588559

[ref30] Park J.-Y.; McKenna G. B. Size and Confinement Effects on the Glass Transition Behavior of Polystyrene/ o -Terphenyl Polymer Solutions. Phys. Rev. B 2000, 61 (10), 6667–6676. 10.1103/PhysRevB.61.6667.

[ref31] Kuon N.; Milischuk A. A.; Ladanyi B. M.; Flenner E. Self-Intermediate Scattering Function Analysis of Supercooled Water Confined in Hydrophilic Silica Nanopores. J. Chem. Phys. 2017, 146 (21), 214501 10.1063/1.4984764. 28595416 PMC5648554

[ref32] Li L.; Zhou D.; Huang D.; Xue G. Double Glass Transition Temperatures of Poly(Methyl Methacrylate) Confined in Alumina Nanotube Templates. Macromolecules 2014, 47 (1), 297–303. 10.1021/ma4020017.

[ref33] Jackson C. L.; McKenna G. B. Vitrification and Crystallization of Organic Liquids Confined to Nanoscale Pores. Chem. Mater. 1996, 8 (8), 2128–2137. 10.1021/cm9601188.

[ref34] Chat K.; Tu W.; Laskowski L.; Adrjanowicz K. Effect of Surface Modification on the Glass Transition Dynamics of Highly Polar Molecular Liquid S-Methoxy-PC Confined in Anodic Aluminum Oxide Nanopores. J. Phys. Chem. C 2019, 123 (21), 13365–13376. 10.1021/acs.jpcc.9b02059.

[ref35] Havriliak S.; Negami S. A Complex Plane Analysis of α-Dispersions in Some Polymer Systems. J. Polym. Sci. Part C Polym. Symp. 1966, 14 (1), 99–117. 10.1002/polc.5070140111.

[ref36] Napolitano S.; Wübbenhorst M. The Lifetime of the Deviations from Bulk Behaviour in Polymers Confined at the Nanoscale. Nat. Commun. 2011, 2 (1), 260 10.1038/ncomms1259.

[ref37] Perez-de-Eulate N. G.; Sferrazza M.; Cangialosi D.; Napolitano S. Irreversible Adsorption Erases the Free Surface Effect on the T g of Supported Films of Poly(4- Tert -Butylstyrene). ACS Macro Lett. 2017, 6 (4), 354–358. 10.1021/acsmacrolett.7b00129. 35610865

[ref38] Tarnacka M.; Madejczyk O.; Kaminski K.; Paluch M. Time and Temperature as Key Parameters Controlling Dynamics and Properties of Spatially Restricted Polymers. Macromolecules 2017, 50 (13), 5188–5193. 10.1021/acs.macromol.7b00616.

[ref39] White R. P.; Lipson J. E. G. Polymer Free Volume and Its Connection to the Glass Transition. Macromolecules 2016, 49 (11), 3987–4007. 10.1021/acs.macromol.6b00215.

[ref40] White R. P.; Lipson J. E. G. How Free Volume Does Influence the Dynamics of Glass Forming Liquids. ACS Macro Lett. 2017, 6 (5), 529–534. 10.1021/acsmacrolett.7b00179. 35610877

[ref41] White R. P.; Lipson J. E. G. Connecting Pressure-Dependent Dynamics to Dynamics under Confinement: The Cooperative Free Volume Model Applied to Poly(4-Chlorostyrene) Bulk and Thin Films. Macromolecules 2018, 51 (20), 7924–7941. 10.1021/acs.macromol.8b01392.

[ref42] Tarnacka M.; Dulski M.; Geppert-Rybczyńska M.; Talik A.; Kamińska E.; Kamiński K.; Paluch M. Variation in the Molecular Dynamics of DGEBA Confined within AAO Templates above and below the Glass-Transition Temperature. J. Phys. Chem. C 2018, 122 (49), 28033–28044. 10.1021/acs.jpcc.8b07522.

[ref43] Adrjanowicz K.; Paluch M. Discharge of the Nanopore Confinement Effect on the Glass Transition Dynamics via Viscous Flow. Phys. Rev. Lett. 2019, 122 (17), 176101 10.1103/PhysRevLett.122.176101. 31107059

[ref44] Tang Q.; Hu W.; Napolitano S. Slowing down of Accelerated Structural Relaxation in Ultrathin Polymer Films. Phys. Rev. Lett. 2014, 112 (14), 1–5. 10.1103/PhysRevLett.112.148306. 24766028

[ref45] Chai Y.; Salez T.; McGraw J. D.; Benzaquen M.; Dalnoki-Veress K.; Raphaël E.; Forrest J. A. A Direct Quantitative Measure of Surface Mobility in a Glassy Polymer. Science (80-.) 2014, 343 (6174), 994–999. 10.1126/science.1244845. 24578574

[ref46] Fakhraai Z.; Forrest J. A. Measuring the Surface Dynamics of Glassy Polymers. Science (80-.) 2008, 319 (5863), 600–604. 10.1126/science.1151205. 18239120

[ref47] Minecka A.; Kaminska E.; Tarnacka M.; Talik A.; Grudzka-Flak I.; Wolnica K.; Dulski M.; Kaminski K.; Paluch M. Conformational Changes Underlying Variation in the Structural Dynamics of Materials Confined at the Nanometric Scale. Phys. Chem. Chem. Phys. 2018, 20 (48), 30200–30208. 10.1039/C8CP06086J. 30489579

[ref48] Kardasis P.; Sakellariou G.; Steinhart M.; Floudas G. Non-Equilibrium Effects of Polymer Dynamics under Nanometer Confinement: Effects of Architecture and Molar Mass. J. Phys. Chem. B 2022, 126 (29), 5570–5581. 10.1021/acs.jpcb.2c03389. 35834553

[ref49] Panagopoulou A.; Napolitano S. Irreversible Adsorption Governs the Equilibration of Thin Polymer Films. Phys. Rev. Lett. 2017, 119 (9), 097801 10.1103/PhysRevLett.119.097801. 28949580

[ref50] Song Z.; Rodríguez-Tinoco C.; Mathew A.; Napolitano S. Fast Equilibration Mechanisms in Disordered Materials Mediated by Slow Liquid Dynamics. Sci. Adv. 2022, 8 (15), 1–8. 10.1126/sciadv.abm7154. PMC9012462 35427165

[ref51] Burroughs M. J.; Napolitano S.; Cangialosi D.; Priestley R. D. Direct Measurement of Glass Transition Temperature in Exposed and Buried Adsorbed Polymer Nanolayers. Macromolecules 2016, 49 (12), 4647–4655. 10.1021/acs.macromol.6b00400.

[ref52] Lunkenheimer P.; Wehn R.; Schneider U.; Loidl A. Glassy Aging Dynamics. Phys. Rev. Lett. 2005, 95 (5), 055702 10.1103/PhysRevLett.95.055702. 16090889

[ref53] Casalini R.; Roland C. M. Aging of the Secondary Relaxation to Probe Structural Relaxation in the Glassy State. Phys. Rev. Lett. 2009, 102 (3), 1–4. 10.1103/PhysRevLett.102.035701. 19257369

[ref54] Kohlrausch R. Ueber Das Dellmann’sche Elektrometer. Ann. der Phys. und Chemie 1847, 148 (11), 353–405. 10.1002/andp.18471481102.

[ref55] Lautrup B. Physics of Continuous Matter; Institute of PHysics Publishing: Bristol, 2005. 10.1201/9781439894200.

