# Supplementary material for: New Insights from Nonequilibrium Kinetics Studies on Highly Polar S-Methoxy-PC Infiltrated into Pores
Source: J Phys Chem Lett. 2022 Nov 3;13(44):10464–70. doi: 10.1021/acs.jpclett.2c02672 (PMC9661534; doi:10.1021/acs.jpclett.2c02672)
Supplement: Supplementary file 1 — jz2c02672_si_001.pdf [file jz2c02672_si_001.pdf]

Supporting Information

New Insights from Non-equilibrium Kinetics Studies on Highly Polar S-methoxy-PC Infiltrated into Pores

Magdalena Tarnacka[†*], Ewa Kamińska[#], Marian Paluch[†], Kamil Kamiński[†]

† *Institute of Physics, University of Silesia in Katowice, 75 Pulku Piechoty 1, 41-500 Chorzow, Poland*

Department of Pharmacognosy and Phytochemistry, Faculty of Pharmaceutical Sciences in Sosnowiec, Medical University of Silesia in Katowice, Jagiellońska 4, 41-200 Sosnowiec, Poland

* Corresponding author: (MT) magdalena.tarnacka@us.edu.pl; magdalena.tarnacka@smcebi.edu.pl

Experimental section

Materials. (S)-(-)-4-methoxymethyl-1,3-dioxolan-2-one (s-methoxy-PC) of purity higher than 98% was supplied from ABCR GmbH and used as received. The chemical structure is presented as the inset in **Figure 1a**. The nanoporous aluminum oxide (AAO) membranes used in this study (supplied from InRedox) are composed of uniaxial channels (open from both sides) with well-defined pore diameter, d. The nanoporous silica membranes were prepared by electrochemical etching of silicon wafers and subsequent thermal oxidation.[1,2] In the experiment, we used both alumina membranes characterized by pore diameter, d = 10 nm, and silica templates of d = 4 nm. The pore size distribution of self-made silica templates was confirmed by measuring the nitrogen adsorption/desorption isotherms; for details, please see ref. 3.

Sample preparation/Infiltration procedure. Before filling, porous membranes were dried in an oven at T = 423 K under vacuum to remove any volatile impurities from the nanochannels. After cooling, they were placed in s-methoxy-PC. The whole system was then maintained at T = 293 K in a vacuum (10^{-2} bar) for t = 24 h to let the compound flow into the nanocavities.

After completing the infiltration process, the surfaces of alumina and silica membranes were dried. The excess sample on the porous surface was removed by use of a metal blade and paper towel. The complete filling was obtained by weighing the templates before and after each infiltration to constant mass. The filling degree reaches ~90%. Those values are calculated taking into account the porosity of membranes and assuming that both (i) the density of the infiltrated material does not change along the pore radius, and finally (ii) the shape of the pore is cylindrical. This parameter depends on the porosity of the applied membranes. Note that we used two types of porous matrices, made of either alumina (AAO) or silica porous templates, which differ in the porosity.

BDS measurements. Isobaric measurements of the complex dielectric permittivity $\varepsilon^*(\omega) = \varepsilon'(\omega) - i\varepsilon''(\omega)$ were carried out using a Novocontrol Alpha dielectric spectrometer over the frequency range from 10^{-2} to 10^6 Hz at ambient pressure. The temperature was maintained with a Quatro Cryosystem using a nitrogen gas cryostat; control was better than 0.1 K. Dielectric measurements of bulk s-methoxy-PC were performed in a parallel-plate cell (diameter: 10 mm, gap: 0.1 mm), see ref. 4. AAO or silica membranes filled with s-methoxy-PC were also placed in a similar capacitor (diameter: 10 mm, the thickness of the membrane: 0.05 mm). Temperature-dependent measurements were carried out in the range T = 180 – 223 K. Time-dependent measurements were carried out in the range T_{anneal} = 187 – 223 K. It should be mentioned that the confined sample is a heterogeneous dielectric consisting of a matrix and an investigated compound. Because the applied electric field is parallel to the long pore axes, the equivalent circuit consists of two capacitors in parallel composed of $\varepsilon^*_{s-methoxy-PC}$ and $\varepsilon^*_{template}$. Thus, the measured total impedance is related to the individual values through $\frac{1}{Z^*_c} = \frac{1}{Z^*_{s-metoxy-PC}} + \frac{1}{Z^*_{template}}$. In this context, the recorded dielectric spectra were recalculated accordingly to the approach presented in ref. 5.

The obtained recalculated dielectric spectra were analyzed by the superposition of Havriliak-Negami (HN) functions[6] to determine the structural relaxation times, τ_α:

$$\varepsilon * (\omega) = \varepsilon_\infty + \frac{\Delta\varepsilon}{[1+(i\omega\tau_{HN})^{\alpha_{HN}}]^{\beta_{HN}}}, \qquad \text{(S1)}$$

where α_{HN} and β_{HN} are the shape parameters representing the symmetric and asymmetric broadening of given relaxation peaks, $\Delta\varepsilon$ is the dielectric relaxation strength, τ_{HN} is the HN relaxation time, ε_0 is the vacuum permittivity, and ω is an angular frequency ($\omega = 2\pi f$). Note that τ_α were estimated from τ_{HN} accordingly to the equation given in ref. 7.

To determine the glass transition temperatures of bulk and confined s-methoxy-PC, the estimated τ_α were fitted by either the Vogel-Fulcher-Tammann (VFT) equation or a combination of the VFT and Arrhenius equations. It should be mentioned that both functions were applied only for the confined systems due to the observed deviation in the slope of $\tau_\alpha(T)$-dependences at $T_{g,interfacial}$. The VFT equation was used only for an accurate determination of a point (temperature) at which the slope changes. The glass transition temperatures of confined samples (in the case of the core molecules) were estimated from the extrapolation of Arrhenius fits. In turn, for the bulk samples, only a single VFT equation was applied[8,9,10]:

$$\tau_\alpha = \tau_\infty exp\left(\frac{D_T T_0}{T - T_0}\right), \qquad \text{(S2)}$$

where τ_∞, D_T and T_0 are the fitting parameters. The Arrhenius equation was used as follows:

$$\tau_\alpha = \tau_\infty exp(\Delta E / k_B T), \qquad \text{(S3)}$$

where k_B is the Boltzmann constant and ΔE is the activation energy. T_g is defined as a temperature at which τ_α = 1 s and the glass transition temperature of core molecules of examined confined materials, determined from the Arrhenius fit's extrapolation, is denoted as $T_{g,core}$.

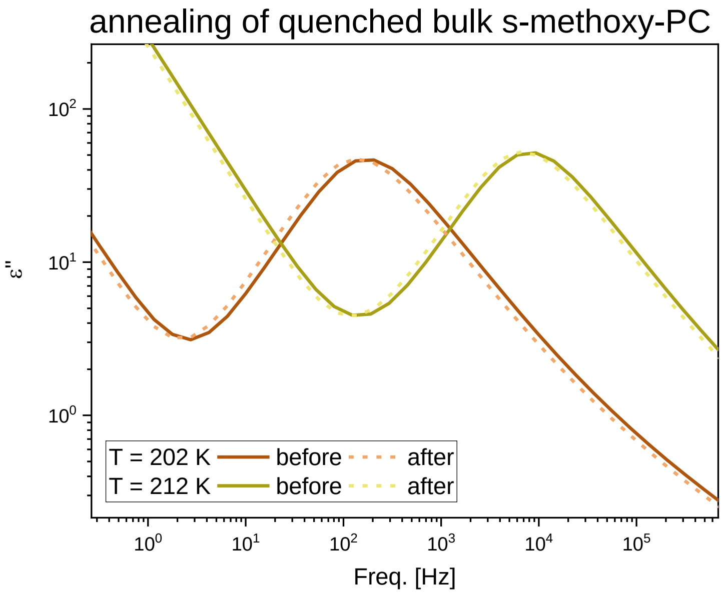

Figure S1. Time evolution of the loss spectra measured for bulk s-methoxy-PC at various T_{anneal} after quenching.

DSC measurements. Calorimetric measurements were carried out by Mettler-Toledo DSC apparatus equipped with a liquid nitrogen cooling accessory and an HSS8 ceramic sensor (heat flux sensor with 120 thermocouples). Temperature and enthalpy calibrations were performed by using indium and zinc standards. The sample was prepared in an open aluminum crucible (40 μL) outside the DSC apparatus. Samples were scanned at various temperatures at a constant heating rate of 10 K/min.

The length scale of the interfacial layer, ζ, can be obtained from the following equation:[11,12]

$$\zeta = \frac{d}{2}\left[1 - \left(1 - \frac{\Delta C_{p.interfacial}}{\Delta C_{p,core} + \Delta C_{p.interfacial}}\right)^{1/2}\right], \qquad \text{(S4)}$$

where $\Delta C_{p.core}$ and $\Delta C_{p.interfacial}$ are the heat capacity changes at $T_{g.core}$ and $T_{g.interfacial}$, respectively. Note that the application of eq S4 requires the following assumptions: (*i*) the volume of the material in the surface layer is proportional to the step change of its heat capacity, (*ii*) the density of the infiltrated material does not change along the pore radius, and finally (*iii*)

the shape of the pore is cylindrical. Note that all calorimetric glass transition temperatures were estimated as the midpoint of the step in specific heat on heating and the heat capacity changes in the heat flow were normalized by the mass of the confined material.

References:

(1) Iacob, C.; Sangoro, J. R.; Papadopoulos, P.; Schubert, T.; Naumov, S.; Valiullin, R.; Kärger, J.; Kremer, F. Charge Transport and Diffusion of Ionic Liquids in Nanoporous Silica Membranes. *Phys. Chem. Chem. Phys.* **2010**, *12* (41), 13798. https://doi.org/10.1039/c004546b.

(2) Kipnusu, W. K.; Kossack, W.; Iacob, C.; Jasiurkowska, M.; Rume Sangoro, J.; Kremer, F. Molecular Order and Dynamics of Tris(2-Ethylhexyl)Phosphate Confined in Uni-Directional Nanopores. *Zeitschrift für Phys. Chemie* **2012**, *226* (7–8), 797–805. https://doi.org/10.1524/zpch.2012.0287.

(3) Talik, A.; Tarnacka, M.; Geppert-Rybczyńska, M.; Hachuła, B.; Bernat, R.; Chrzanowska, A.; Kaminski, K.; Paluch, M. Are Hydrogen Supramolecular Structures Being Suppressed upon Nanoscale Confinement? The Case of Monohydroxy Alcohols. *J. Colloid Interface Sci.* **2020**, *576*, 217–229. https://doi.org/10.1016/j.jcis.2020.04.084.

(4) Jedrzejowska, A.; Ngai, K. L.; Paluch, M. Modifications of Structure and Intermolecular Potential of a Canonical Glassformer: Dynamics Changing with Dipole−Dipole Interaction. *J. Phys. Chem. A* **2016**, *120* (44), 8781–8785. https://doi.org/10.1021/acs.jpca.6b08128.

(5) Tu, W.; Richert, R.; Adrjanowicz, K. Dynamics of Pyrrolidinium-Based Ionic Liquids under Confinement. I. Analysis of Dielectric Permittivity. *J. Phys. Chem. C* **2020**, *124* (9), 5389–5394. https://doi.org/10.1021/acs.jpcc.0c00156.

(6) Havriliak, S.; Negami, S. A Complex Plane Analysis of α-Dispersions in Some Polymer Systems. *J. Polym. Sci. Part C Polym. Symp.* **2007**, *14* (1), 99–117. https://doi.org/10.1002/polc.5070140111.

(7) *Broadband Dielectric Spectroscopy*; Kremer, F., Schönhals, A., Eds.; Springer Berlin Heidelberg: Berlin, Heidelberg, 2003. https://doi.org/10.1007/978-3-642-56120-7.

(8) Vogel, H. Temperatura Bhangigkeitgesetz Der Viskosität von Flüssigkeiten. *J. Phys. Z.* **1921**, *22*, 645–646.

(9) Fulcher, G. S. Analysis of Recent Measurements of the Viscosity of Glasses. *J. Am. Ceram. Soc.* **1925**, *8*, 339–355.

(10) Tammann, G.; Hesse, W. Die Abhängigkeit Der Viscosität von Der Temperatur Bie Unterkühlten Flüssigkeiten. *Z. Anorg. Allg. Chem.* **1926**, *156*, 245–257.

(11) Jackson, C. L.; McKenna, G. B. Vitrification and Crystallization of Organic Liquids Confined to Nanoscale Pores. *Chem. Mater.* **1996**, *8* (8), 2128–2137. https://doi.org/10.1021/cm9601188.

(12) Park, J.-Y.; McKenna, G. B. Size and Confinement Effects on the Glass Transition Behavior of Polystyrene/ o -Terphenyl Polymer Solutions. *Phys. Rev. B* **2000**, *61* (10),

6667–6676. https://doi.org/10.1103/PhysRevB.61.6667.
